# Improved Variant Calling Accuracy by Merging Replicates in Whole-Exome Sequencing Studies

**DOI:** 10.1155/2014/319534

**Published:** 2014-08-04

**Authors:** Yanfeng Zhang, Bingshan Li, Chun Li, Qiuyin Cai, Wei Zheng, Jirong Long

**Affiliations:** ^1^Division of Epidemiology, Department of Medicine, Vanderbilt Epidemiology Center, Vanderbilt-Ingram Cancer Center, Vanderbilt University School of Medicine, Nashville, TN 37203, USA; ^2^Department of Molecular Physiology and Biophysics, Center for Human Genetics Research, Vanderbilt University School of Medicine, Nashville, TN 37232, USA; ^3^Department of Biostatistics, Vanderbilt University School of Medicine, Nashville, TN 37232, USA

## Abstract

In large scale population-based whole-exome sequencing (WES) studies, there are some samples occasionally sequenced two or more times due to a variety of reasons. To investigate how to efficiently utilize these duplicated sequencing data, we conducted comprehensive evaluation of variant calling strategies. 92 samples subjected to WES twice were selected from a large population study. These 92 duplicated samples were divided into two groups: group H consisting of the higher sequencing depth for each subject and group L consisting of the lower depth for each subject. The merged samples for each subject were put in a third group M. Using the GATK multisample toolkit, we compared variant calling accuracy among three strategies. Hierarchical clustering analysis indicated that the two replicates for each subject showed high homogeneity. The comparative analyses on the basis of heterozygous-homozygous ratio (Hete/Homo), transition-transversion ratio (Ti/Tv), and overlapping rate with the 1000 Genomes Project consistently showed that the data quality of the SNPs detected from the M group was more accurate than that of SNPs detected from the H and L groups. These results suggested that merging homogeneous duplicated exomes instead of using one of them could improve variant calling accuracy.

## 1. Introduction

Next generation sequencing technologies generate huge amount of data in a single experimental run and provide a revolutionary tool for various genomics studies [1]. Primarily designed to capture the intended coding variants [2–4], whole-exome sequencing (WES) has been commonly used in basic and translational research, including investigation of diversity and demographic history in human populations [5–8], identification of etiological variants [9–13], cross-species genome comparison [14, 15], and even phylogenetic estimation [16].

Among currently available exome enrichment platforms, some platforms such as Illumina TruSeq were also designed to capture a portion of noncoding regions, including untranslated regions (UTRs) and intronic regions [17]. In addition, we previously found that a significant amount of DNA fragments from WES falls outside the intended regions and a large portion of these fragments is of high quality [18].

In large WES studies, some samples are occasionally sequenced twice or even more times due to a variety of reasons, for example, insufficient coverage in the first experiment, sample duplication, and the rest. It is challenging how to best utilize these duplicated exomes for SNP discovery and genotype calling, especially with batch effects taken into consideration. In the present study, we systematically evaluated SNP detection performance of three strategies to utilize the duplicated exome data of 92 subjects, only using the data with higher depth, only using the data with lower depth, and using the merged data from technical replicates.

## 2. Materials and Methods

### 2.1. Sample Collection

The subjects in this study were a subset of participants in the Shanghai Breast Cancer Study (SBCS), which was a population-based breast cancer case control study. Details of the study have been described elsewhere [19, 20]. In brief, the SBCS is a population-based, case control study including 3,448 breast cancer cases and 3,474 controls. Of these, 92 subjects (51 cases and 41 controls) were included in the current investigation. Genomic DNA from buffy coat was extracted using QIAmp DNA kit (Qiagen, Valencia, CA) following the manufacture's protocol. Approval of the study was granted by the relevant institutional review boards in both China and the United States.

### 2.2. Exome Sequencing and Variant Calling

**Figure 1:**
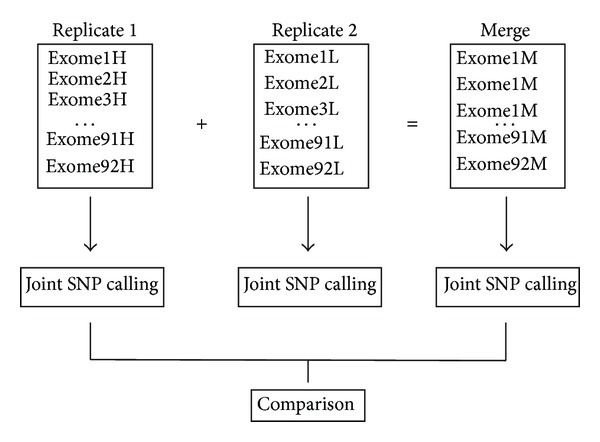
Schematic of SNP calling strategies in this study. For 92 subjects with duplicates, we divided them into two groups (replicate 1 and replicate 2) according to mean sequencing depth across the target regions. The replicate 1 group is comprised of exomes with the relative high mean depth (marked with Exome1H to Exome92H). The remainders with the relative low depth are grouped as replicate 2. Merging BAM files from the two duplicates generates a third group, referred to as the merged group flagged with Exome1M to Exome92M.

For each of 92 subjects, one sequencing library was constructed for genomic DNA captured on the Illumina TruSeq platform according to the manufacturer's instructions and subjected to 75 or 100 bp paired end sequencing twice on the Illumina HiSeq instrument. For each exome, raw reads in FASTQ format were aligned to the human reference genome (hg19) using Burrows-Wheeler Aligner (BWA, v0.5.9) in default parameters [21]. Mapped data were then processed and sorted using SAMtools [22]. Local realignment, PCR duplicates removal, and base quality score recalibration (BQSR) were performed using Genome Analysis toolkit (GATK) to generate BAM files [23]. A total of 184 BAM files were then divided into two groups, high depth (H) and low depth (L) from each subject, based on the mean depth across on-target regions. We also merged the two BAM files for each subject into a single BAM file and combined 92 merged BAM files as a third group (M). We called SNPs within each group (H, L, and M) via the GATK multisample strategy [24] (Figure 1). Then, we conducted the variants filtering as follows: (1) ≥ 3 SNPs detected within 10 bp distance; (2) > 10% alignments mapped ambiguously; (3) SNPs having a quality score < 50; (4) variant confidence/quality by depth < 1.5; (5) strand bias score calculated by GATK > −1.

### 2.3. Statistics of Exome-Sequencing Performance

The metric of RPKM (reads per kilobase and million mapped reads) is a commonly used method for RNA-sequencing data normalization. Recently, the RPKM was also used to evaluate the performance of exome sequencing [25]. In this study, the RPKM was employed and calculated as the number of reads aligned on the target region per kilobase of target sequence divided by the total number of mapped reads. The reads aligned to the 100 bp upstream or downstream of the target regions were also included in the PRKM statistic [25]. The Euclidean distance among the 184 exomes was calculated based on the RPKM value. We conducted the unsupervised hierarchical clustering analysis using R programming language (version 2.15.1) to evaluate batch effects among 92 replicated sequencing experiments.

### 2.4. SNP Detection Performance

We compared SNP detection performance for each of three SNP calling strategies from the following aspects: (1) the total number of SNPs observed; (2) the ratio of heterozygous genotypes to nonreference homozygous genotypes (Hete/Homo), which is expected to be close to 1.5 on genomic scale [26, 27]; (3) the transition-transversion ratio (Ti/Tv), which is expected to be ~2 across the whole genome and ~3 across the exon regions [18, 27]; (4) the overlapping rate between the SNPs uniquely detected within each group with the SNPs observed in the 1000 Genomes (1 KG) Project [28]. If a closer approximation to the empirically expected value as well as a higher overlapping rate is observed, it generally manifests lower false discovery rate. The evaluation was inherently stratified, according to the locations of SNPs, to on-target regions and off-target regions. All analyses were achieved by a series of custom Perl scripts.

## 3. Results 

### 3.1. Data Generation between the Duplicates

**Figure 2:**
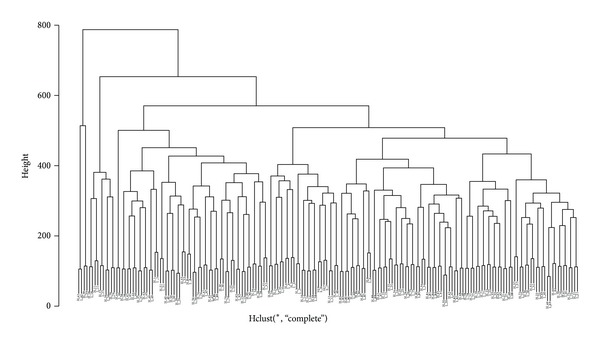
Hierarchical clustering of replicated exomes of 92 subjects. The pairwise distance is based on the RPKM matrix for each exome. Duplicates within each subject are flagged with prefix of H or L.

Table 1 summarizes the statistics for the exome sequencing data. We obtained an average of 64.0 and 57.2 million reads per exome, with 43.4 and 36.0 mean depths across the target regions, for the H and L group, respectively (see Supplementary Table  1 in Supplementary Material available online at http://dx.doi.org/10.1155/2014/319534). On average, 98.23% and 98.65% of the reads were aligned to the human reference genome, and 49.70% and 49.11% were mapped to the target regions, in the H and L groups, respectively. Approximately 86.16% and 86.14% of the reads in the H and L groups had mapping quality ≥ 20, respectively. Overall, the comparative results showed the high homogeneity between the duplicates for each subject (Supplementary Table  1). Furthermore, the hierarchical clustering result based on PRKM values also showed that the two replicates for each subject were closely clustered with each other (Figure 2). These results suggest there is low heterogeneity between the replicated experiments.

### 3.2. SNP Detection Performance

**Figure 3:**
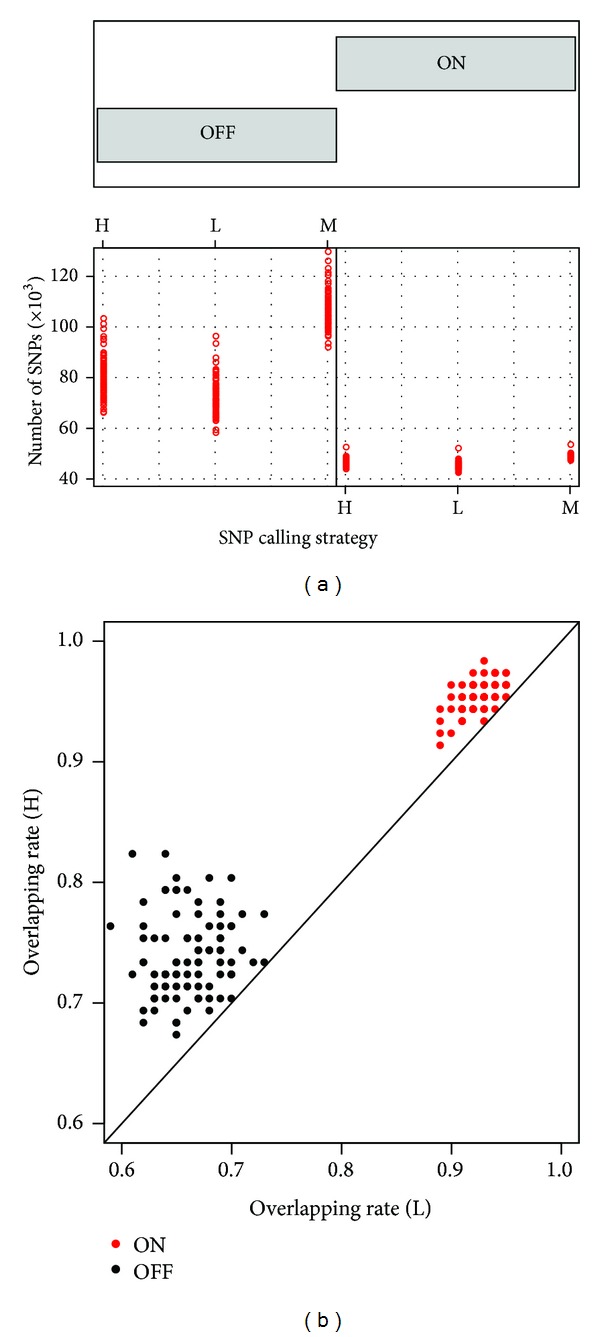
Comparison of SNPs identified across the on-target and off-target regions in three groups (M, L, and H). (a) Each red circle denotes the number of SNPs detected in the three groups within two regions (ON and OFF). (b) Overlapping rate of the SNPs detected in the H (*y*-axis) and L (*x*-axis) groups with the M group. Each dot denotes the overlapping rate of SNPs across the ON (red) and OFF (black) regions in each corresponding exome. ON and OFF represent the on-target and off-target regions, respectively; M, L, and H represent the merged, low depth, and high depth groups, respectively.

First, we compared the number of SNPs called from different strategies.  Figure 3 summarizes the number of SNPs detected in each exome. For the target regions, the average number of SNPs observed in each exome was similar across the three groups, with 46,860, 44,806, and 43,664 for the M, H, and L groups, respectively (Figure 3(a)). Among these SNPs across the TruSeq target regions detected in the M group, 95.0% and 92.5% of them were also observed in the H and L groups, respectively. Approximately 94.9% of the SNPs observed in the H group were also detected in the L group. Similarly, 94.8% of the SNPs in the L group were also detected in the H group.

For those SNPs detected across the off-target regions, the M group detected far more SNPs than the other two groups with 105,154 SNPs versus 78,745 and 70,766 per subject for H and L, respectively (Figure 3(a) and Supplementary Table  2). Almost all SNPs (97.9%) detected in the H and L groups were also found in the M group, while only 73.5% and 66.2% of SNPs detected in the M group could be identified in the H and L groups, respectively (Figure 3(b)). Together, an average of 1,480, 1,246, and 23,397 SNPs across 92 subjects was uniquely detected in the H, L, and M group, respectively (Supplementary Table  2), where over 80% of the SNPs within each group were located in the off-target regions.

**Figure 4:**
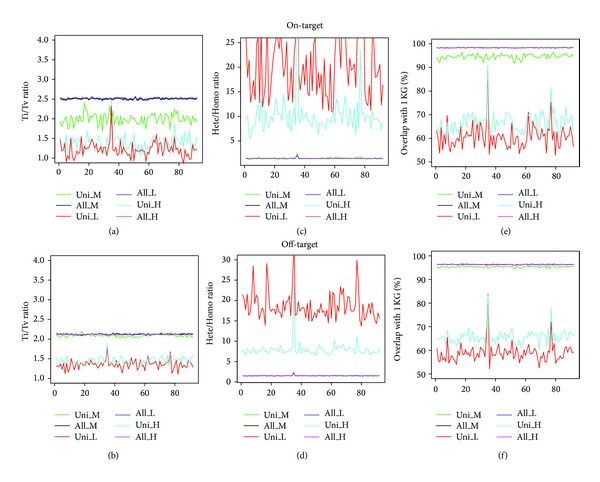
Comparison of the accuracy of SNPs detected by three strategies. The upper panel is plotted for SNPs across the on-target regions; the lower panel is for the off-target SNPs. From the left to right in each panel are the Ti/Tv ratio ((a) and (b)), Hete/Homo ratio ((c) and (d)), and overlapping rate with SNPs observed in the 1 KG project ((e) and (f)). Uni_M, Uni_L, and Uni_H represent the number of SNPs uniquely identified by the merged, low depth, and high depth strategies, respectively; All_M, All_L, and All_H denote the total number of SNPs identified by the merged, low depth, and high depth strategies, respectively. The *x*-axis represents each of 92 exome-seq samples.

We then examined the SNP calling accuracy. The M group also showed better performance than the H and the L groups. For SNPs located in the target regions and uniquely observed within each group, the average Ti/Tv ratios were 2.00 ± 0.13 (mean ± SD), 1.39 ± 0.21, and 1.22 ± 0.19 for the M, H, and L groups, respectively (Figure 4(a)). For SNPs located in the off-target regions, the corresponding Ti/Tv ratios were 2.09 ± 0.03, 1.46 ± 0.09, and 1.35 ± 0.11 for the M, H, and L groups, respectively (Figure 4(b)).

The Hete/Homo ratio was another parameter to evaluate the genotype quality with the expected value of 1.5 on a genomic scale [26, 27]. Among the SNPs uniquely observed in the M group, the average Hete/Homo ratio was 1.51 and 1.52 in the on-target region and off-target regions, respectively (Figures 4(c) and 4(d)). However, the Hete/Homo ratios were much larger for SNPs uniquely observed in the H and L groups, being 10.45 and 22.43 across the on-target regions and 7.93 and 18.92 across the off-target regions. Among the SNPs uniquely discovered in the M group, 94.4% were included in the 1 KG project. However, only 65.7% and 59.3% of the SNPs uniquely called in the H and L groups were overlapped with the SNPs in the 1 KG project (Figures 4(e) and 4(f)).

**Figure 5:**
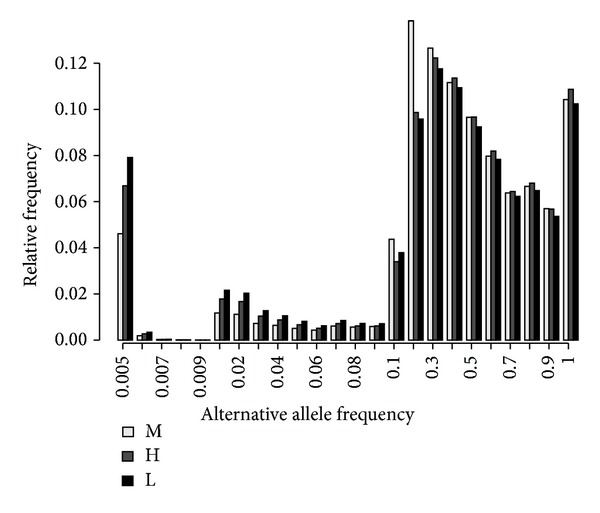
Alternative allele frequency distribution of all SNPs called in each of the three groups, H, L, and M.

We finally compared the allele frequency of variants called by three approaches.  Figure 5 shows the alternative allele (relative to the hg19 reference genome) frequency (AF) distribution among the SNPs observed in these three groups. For alternative alleles with AF ≥ 0.1, it showed that the allele frequency spectrum among three groups was overall identical, despite a slight difference in the range of 0.1-0.2. However, for low-frequency alleles (AF ≤ 0.05), we found the allele frequency is significantly lower (two-tailed Fisher's exact test, *P* < 0.001) in the M group (Figure 5), almost up to 2 times lower than that in the L group. When the frequency of uniquely observed SNPs was considered, similar patterns were observed (Supplementary Figure  1), suggesting that merging duplicated exome-seq data within each subject would reduce false discovery rates for rare variants.

## 4. Discussion

In this study, we comprehensively investigated the variant calling based on three strategies to utilize the duplicated WES data from 92 subjects. From the aspects of the number of high quality variants, Hete/Homo ratio, Ti/Tv ratio, and overlapping rate with the 1 KG, our comparative analyses indicate that the M strategy (merging duplicates into one) is markedly superior to the other two approaches, especially for identifying variants located in the off-target regions.

The Ti/Tv ratio is a critical metric for assessing the specificity of SNP calling [23]. The empirical Ti/Tv ratios are ~2.0 for genome-wide variants and ~3.0–3.3 for coding variants [18, 27]. Typically, the Ti/Tv ratio is lower in the newly discovered SNPs than that in known SNPs because of a combination of residual false positives, a relative deficit of transitions due to sequencing context bias, and an apparently higher transition ratio at lower frequency variation [23]. The Ti/Tv ratio for SNPs uniquely called in the M group was much closer to expected value, suggesting the higher confidence of these SNPs than those uniquely called in H/L groups. The Hete/Homo ratio, an average of ~1.5 on the genomic scale [26, 27], is another metric for assessing the SNP calling accuracy. The observed Hete/Homo ratio was 1.51 ± 0.13 (± s.d.) for these SNPs uniquely observed in the M group. In addition, the overlapping rate between the SNPs uniquely detected in the M group and the 1 KG project was much higher than that in the L and H groups. These results are consistent with Liu et al.'s report that multiple-sample calling by GATK pipelines increased the sensitivity of variants calling [29].

Although the aim of WES is to identify SNPs located in the coding regions, not all coding variants could be equally discovered. There are diverse reasons, including high or low GC contents in the target regions, uneven DNA capture, and sequencing depth [30–32]. Besides improving technical issues, the maximal utilization of WES data, particularly in the noncoding regions, is also of great interest. The enrichment of SNPs in the off-target regions might be related to the capturing probe design and hybridization steps. Especially, increasing the sequencing depth (equivalent to the merged strategy) per sample may help to identify more off-target SNPs. SNPs in off-target regions are reported in multiple capture platforms, including Illumina, NimbleGen, and Agilent platforms [17, 18]. However, SNPs located in off-target regions exhibit more variability in the number of variants and data quality. This suggests that more consideration should be taken into account when calling SNPs across the off-target regions. In the present study, we found that merging BAM files from duplicated sequencing data could greatly enhance the detection of high quality SNPs in off-target regions.

Merging two or more replicated sequencing data could improve SNP calling accuracy, as this strategy is to a large extent similar to the addition of sequencing depth. However, there is a prerequisite. It needs to assess the heterogeneity between replicates within each subject before merging them. Multiple measurements, including on-target region mapping rate, low quality read rate, the fraction of four bases, and genome coverage, could be used for evaluation. We also propose that the hierarchical clustering based on the RPKM statistic is a robust evaluation for quality control in the WES performance [25] as the RPKM statistic does for RNA-sequencing data [33, 34]. In addition, due to the fact that replicated experiments are generated from the same sequencing library preparation, which shows the low batch problem, it is unclear whether merging replicated samples prepared from the different sequencing libraries also reaches this conclusion. As Leek et al. reported [35], large batch effects are associated with DNA preparation group and processing date. Therefore, if the two duplicates within each subject show remarkable disparity in the quality control, it is cautious to use merging strategy for further analysis.

In summary, our investigation indicates that merging the low heterogeneous duplicated WES data within each subject into a single sample and then conducting SNP calling are a reasonable strategy to discover variants from the next generation sequencing technology.

## Supplementary Material

Table S1: Data production by 92 duplicated WES subjects.
Table S2: Number of variants observed across the on-target and off-target regions.
Figure S1: Alternative allele frequency distribution of SNPs uniquely called in H and M.

## Figures and Tables

**Table 1:** Summary of WES samples divided into two groups (H and L).

Group	Total reads (×10^6^)	Mapping rate (%)	Target mapping (%)	Mean depth^a^	Mapping rate with MQ < 10 (%)^b^	Mapping rate with MQ ≥ 20 (%)^b^
H	64.0 (49.2–97.9)	98.2 (95.9–99.2)	49.7 (46.6–55.4)	43.4 (34.2–65.8)	10.4 (9.5–11.3)	86.2 (84.0–87.7)
L	57.2 (40.3–79.3)	98.7 (97.6–99.2)	49.1 (44.5–55.0)	36.0 (29.5–53.0)	10.8 (9.6–12.3)	86.1 (84.4–87.6)

^a^Mean depth across target regions.

^b^MQ denotes mapping quality.

Note that the range of values is shown in the parenthesis.

## Abbreviations

GATK: Genome analysis toolkit
Hete/Homo: Heterozygous-homozygous ratio
Ti/Tv: Transition-transversion ratio
WES: Whole-exome sequencing
SBCS: Shanghai breast cancer study
H: High depth
L: Low depth
M: Merged
RPKM: Reads per kilobase and million mapped reads
1 KG: 1000 genomes.
