# Supplementary figures and images for: Improved Variant Calling Accuracy by Merging Replicates in Whole-Exome Sequencing Studies
Source: Biomed Res Int. 2014 Aug 4;2014:319534. doi: 10.1155/2014/319534 (PMC4137624; doi:10.1155/2014/319534)

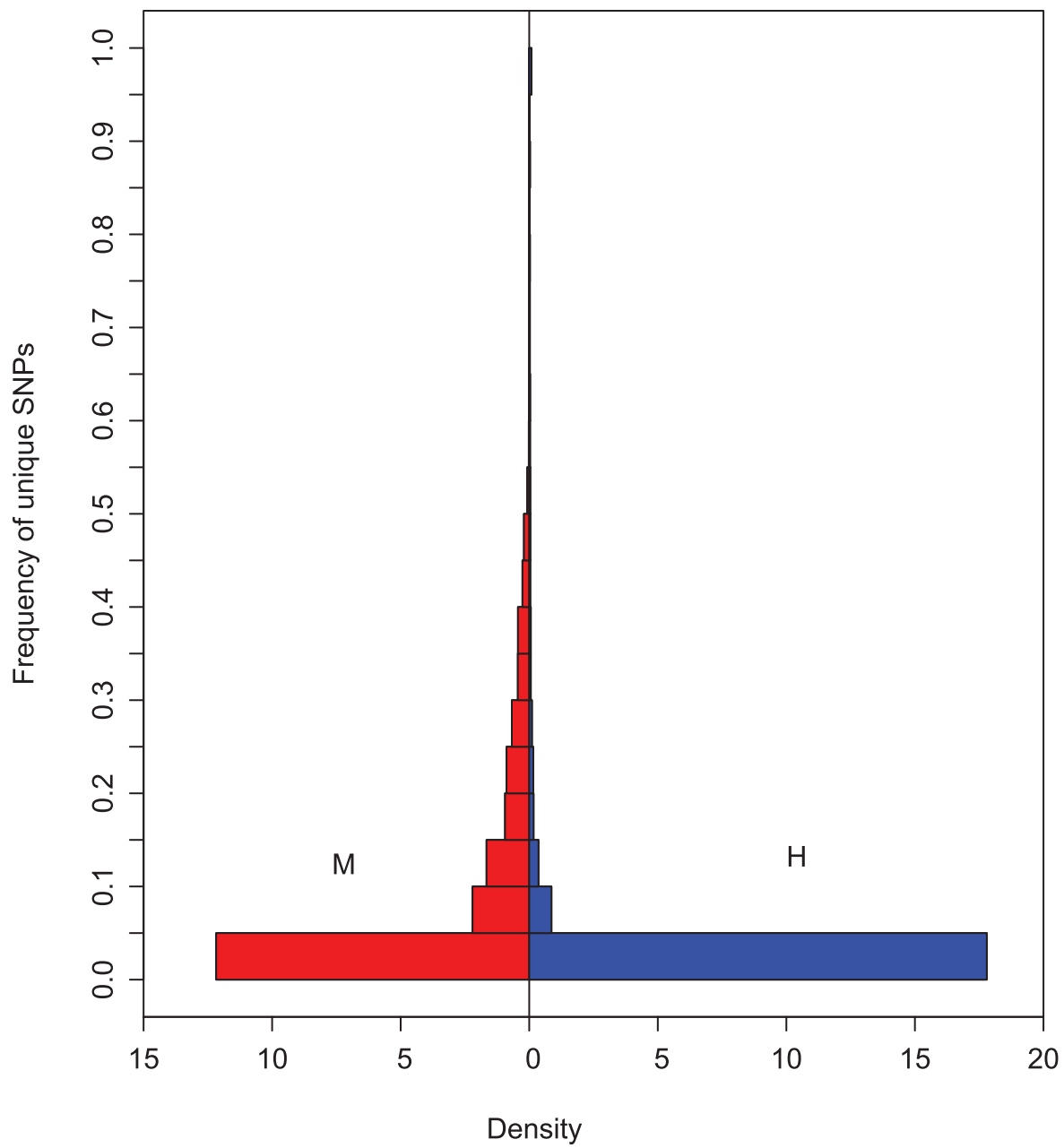

Supplement: Supplementary file 2 [file 319534.f2.pdf]
